# The naturally competent strain *Streptococcus thermophilus* LMD-9 as a new tool to anchor heterologous proteins on the cell surface

**DOI:** 10.1186/1475-2859-13-82

**Published:** 2014-06-05

**Authors:** Xavier Lecomte, Valérie Gagnaire, Valérie Briard-Bion, Julien Jardin, Sylvie Lortal, Annie Dary, Magali Genay

**Affiliations:** 1 Unité de Recherche Animal et Fonctionnalités des Produits Animaux, Equipe Protéolyse et Biofonctionnalité des Protéines et des Peptides, Université de Lorraine, Vandœuvre-lès-Nancy F-54506, France; 2 INRA, UR AFPA Unité Sous Contrat 340, Vandœuvre-lès-Nancy F-54506, France; 3 UMR 1253, INRA, Science et Technologie du Lait et de lŒuf, Rennes, France; 4 UMR 1253, Agrocampus Ouest, Science et Technologie du Lait et de lŒuf, Rennes, France

**Keywords:** Heterologous expression, Secretion, Cell-wall anchored protein, *Streptococcus thermophilus* LMD-9, Cell envelope proteinase (CEP), PrtS, PrtH, *Lactobacillus helveticus* CNRZ32 CIRM-BIA 103

## Abstract

**Background:**

From fundamental studies to industrial processes, synthesis of heterologous protein by micro-organisms is widely employed. The secretion of soluble heterologous proteins in the extracellular medium facilitates their recovery, while their attachment to the cell surface permits the use of the recombinant host cells as protein or peptide supports. One of the key points to carry out heterologous expression is to choose the appropriate host. We propose to enlarge the panel of heterologous secretion hosts by using *Streptococcus thermophilus* LMD-9. This lactic acid bacterium has a generally recognised as safe status, is widely used in the manufacture of yogurts, fermented milks and cheeses, and is easy to transform by natural competence. This study demonstrates the feasibility of secretion of a heterologous protein anchored to the cell surface by *S. thermophilus*. For this, we used the cell envelope proteinase (CEP) PrtH of *Lactobacillus helveticus* CNRZ32 CIRM-BIA 103.

**Results:**

Using *S. thermophilus* LMD-9 as the background host, three recombinant strains were constructed: i) a negative control corresponding to *S. thermophilus* PrtS^-^ mutant where the *prtS* gene encoding its CEP was partially deleted; ii) a PrtH^+^ mutant expressing the *L. helveticus* PrtH pro-protein with its own motif (S-layer type) of cell-wall attachment and iii) a PrtH^+^WANS mutant expressing PrtH pro-protein with the LPXTG anchoring motif from PrtS. The *PrtH*^*+*^ and *PrtH*^*+*^*WANS* genes expression levels were measured by RT-qPCR in the corresponding mutants and compared to that of *prtS* gene in the strain LMD-9. The expression levels of both fused *prtH* CEPs genes, regardless of the anchoring motif, reached up-to more than 76% of the wild-type *prtS* expression level. CEPs were sought and identified on the cell surface of LMD-9 wild-type strain, PrtH^+^ and PrtH^+^WANS mutants using shaving technique followed by peptide identification with tandem mass spectrometry, demonstrating that the heterologous secretion and anchoring of a protein of more than 200 kDa was efficient. The anchoring to the cell-wall seems to be more efficient when the LPXTG motif of PrtS was used instead of the S-layer motif of PrtH.

**Conclusions:**

We demonstrated *S. thermophilus* LMD-9 could heterologously secrete a high molecular weight protein and probably covalently anchor it to the cell-wall.

## Background

Heterologous protein secretion is being increasingly employed to produce proteins on different living supports. Genetic tools have therefore been developed to present a protein or a peptide specifically on the surface of a microbial vector to study its structure, conformation, activity and/or interactions with its environment. Studies could be conducted in various conditions such as in a culture medium, in dairy matrix, or *in vivo,* for immune response (vaccination) or physiological effects in gastro intestinal tract [1,2]. In order to secrete heterologous proteins in the extracellular medium or anchor them to the cell-wall, the choice of the host is crucial and a panel of transformable species with their advantages and disadvantages is currently available [1]. The gram negative bacterium *Escherichia coli* is the most employed for intracellular heterologous expression, while the gram positive bacterium *Bacillus subtilis* is preferentially chosen for heterologous secretion [1,3]. *B. subtilis* also displays the advantage to be naturally competent. Indeed, it is possible in a one-step plasmid-free transformation to introduce into the cell and integrate in the chromosome by homologous recombination a foreign DNA. Lactic acid bacteria (LAB) have recently been used as alternative expression hosts to *B. subtilis*[4]. Among them, *Lactococcus lactis* presents several advantages, including many tools for genetic modifications, efficient protein secretion capability [4] and only few predicted cell surface proteins [5]. Furthermore, this bacterium possesses only one surface housekeeping protease, HtrA, able to degrade abnormal exported proteins and this protease gene can be inactivated to prevent the degradation of heterologously expressed proteins [4]. Finally, as this species has a generally recognised as safe (GRAS) status and is widely present in dairy products, it could be employed to study proteins behaviour in food (*in situ*) or *in vivo* which is not the case for *B. subtilis.* However, *Lc. lactis* still lacks the natural competence [6] in contrast to other food grade LAB [7] such as *Streptococcus thermophilus*.

*S. thermophilus* has a GRAS status and is widely used for its high acidification ability, in dairy products. This is one of the two dairy starters of yogurt and its recovery in faeces of human volunteers consuming yogurt has definitively established that this bacterium is capable to remain alive during its transit through the digestive tract [8,9]. Further, the production of active β-galactosidase in the second half of the small intestine by the strain FB13 further suggests that *S. thermophilus* remains metabolically active during its digestive transit. This could explain the improvement of lactose digestion observed in intolerant patients after yogurt consumption [9,10].

Previous works demonstrated that *S. thermophilus* was able to express intracellular heterologous proteins. Mutants were obtained by electrotransformation and plasmids were used as gene vectors to produce intracellular heterologous proteins. For example, green fluorescent protein [11], *Streptomyces* cholesterol oxidase [12] and glutamate decarboxylase [13] were heterologously produced in *S. thermophilus* cytoplasm. The recent discovery of natural competence and its mechanisms in some *S. thermophilus* strains [14-16] allowed the introduction of heterologous genes in the bacterial cell by natural competence and their chromosomal insertion by double cross-over recombination. Since then, natural competence was used to insert linear DNA fragments corresponding to reporter genes such as the luciferase genes *luxAB*[17] and the antibiotic resistant genes, i.e. *cat* for chloramphenicol [17], *aphA3* for kanamycin or *ery* for erythromycin [18] which were intracellularly expressed. Now the challenge is to produce mutants able to export proteins on the cell surface and in this context, *S. thermophilus* LMD-9 could be a good candidate. Indeed, on the basis of its genome sequence, it is expected that this bacterium would display 2/3 less cell surface proteins than *Lc. lactis*, which already have few proteins present at the surface [5]. A single cell envelope proteinase (CEP), named PrtS, with high molecular weight is predicted to be covently anchored onto the cell-wall by action of the sortase SrtA [19]. The secretion system exporting this CEP as well as the sortase SrtA could then be good candidates to secrete in the medium heterologous proteins of various molecular weights and to covalently anchor them onto the cell-wall. Further, as in the case of *Lc. lactis,* the presence of a unique housekeeping protease gene, *htrA*, is an advantage compared to *B. subtilis* that possesses multiple housekeeping proteases able to degrade the exported proteins at the surface [3], and *htrA* of *S. thermophilus* can be deleted to avoid protein degradation. Finally, *S. thermophilus* has been detected in human gut by metagenomic sequencing [20], further make possible the use of this bacterium in *in vivo* studies. Therefore, it could be interesting to further develop genetic tools to produce heterologous proteins on the cell-wall of *S. thermophilus* LMD-9 strain.

To make the proof of concept that *S. thermophilus* can be used as a tool for the heterologous secretion of cell-wall anchored proteins, we decided to heterologously express the *prtH* gene of *L. helveticus* CNRZ32 in *S. thermophilus* LMD-9. Indeed, one of the main topics of our laboratories is the study of the activity and specificity of CEPs of lactic acid bacteria, particularly those of *S. thermophilus* and *L. helveticus*. The choice of this protease seemed coherent to us knowing that: i) *S. thermophilus* and *L. helveticus* CEP genes present similarities in the codon usage; ii) both bacteria are used as co-starters in the manufacturing of Swiss-type cheeses and thus grow in the same environment (milk medium, temperature, pH …), thereby eliminating a potential influence of the environment on the conformation and activity of PrtH; iii) *S. thermophilus* possesses an active proteolytic system and so anything that seems necessary for the expression and activity of a protease; iv) proteolytic activity would be easy to detect as it is known to confer a growth advantage compared to strains that do not secrete a functional CEP [21,22]. Finally, this experiment may allow us to elucidate the specific role of PrtH in the proteolytic activity of *L. helveticus* CNRZ32 since four CEP genes were identified in its genome sequence designated as *prtH*, *prtH2*, *prtH3* and *prtH4* and the role of each CEP has not been defined independently yet [23,24].

## Results

### Strategy of the mutant constructions for the heterologous expression of PrtH on the cell-wall of *S. thermophilus* LMD-9

**Figure 1:**
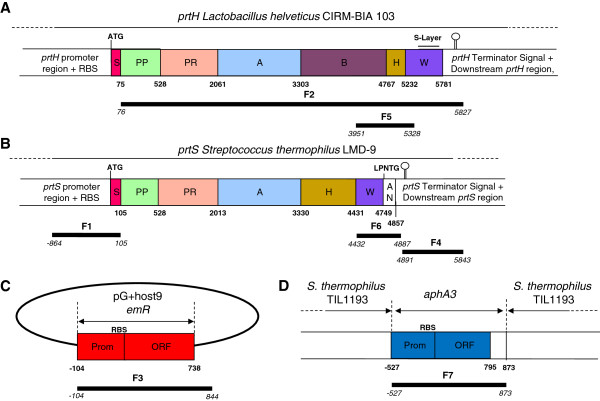
**Schematic representation of DNA matrix and fragments amplified for mutant constructions.** Black bars (F1 to F7) represent the fragments amplified from *prtS*, *prtH*, *emR* and *aphA3* genes used to produce mutants expressing a PrtH fusion protein. Numbers refer to the last nucleotide of a domain (bold characters) or fragment limits (italic characters), starting from the adenosine nucleotide of the start codon of the ORF. The proteinase genes *prtH***(A)** of *Lactobacillus helveticus* CNRZ32 CIRM-BIA 103 [this study, [28] and *prtS***(B)** of *Streptococcus thermophilus* LMD-9 [this study, [25] are represented with their domains: upstream regions contain promoter and ribosome binding site (RBS), followed by S (signal sequence domain), PP (propeptide domain), PR (catalytic domain), A (A domain), B (B domain) in *prtH*, H (helix domain), W (cell-wall spacer domain) containing in *prtH* a 303 nucleotides S-layer attachment domain (small bar) and AN (anchor domain) in *prtS* with the LPNTG anchoring site. The downstream regions with the *prtH* and *prtS* terminator signals are also represented [22,28]. The erythromycin resistance gene (*emR*) is presented in the pG+host9 plasmid **(C)** with its promoter (Prom) and its ORF [48]. The kanamycin resistance gene (*aphA3*) inserted in *S. thermophilus* LMD-9 TIL1193 chromosome **(D)** is presented with its promoter (Prom) and its ORF [18].

**Figure 2:**
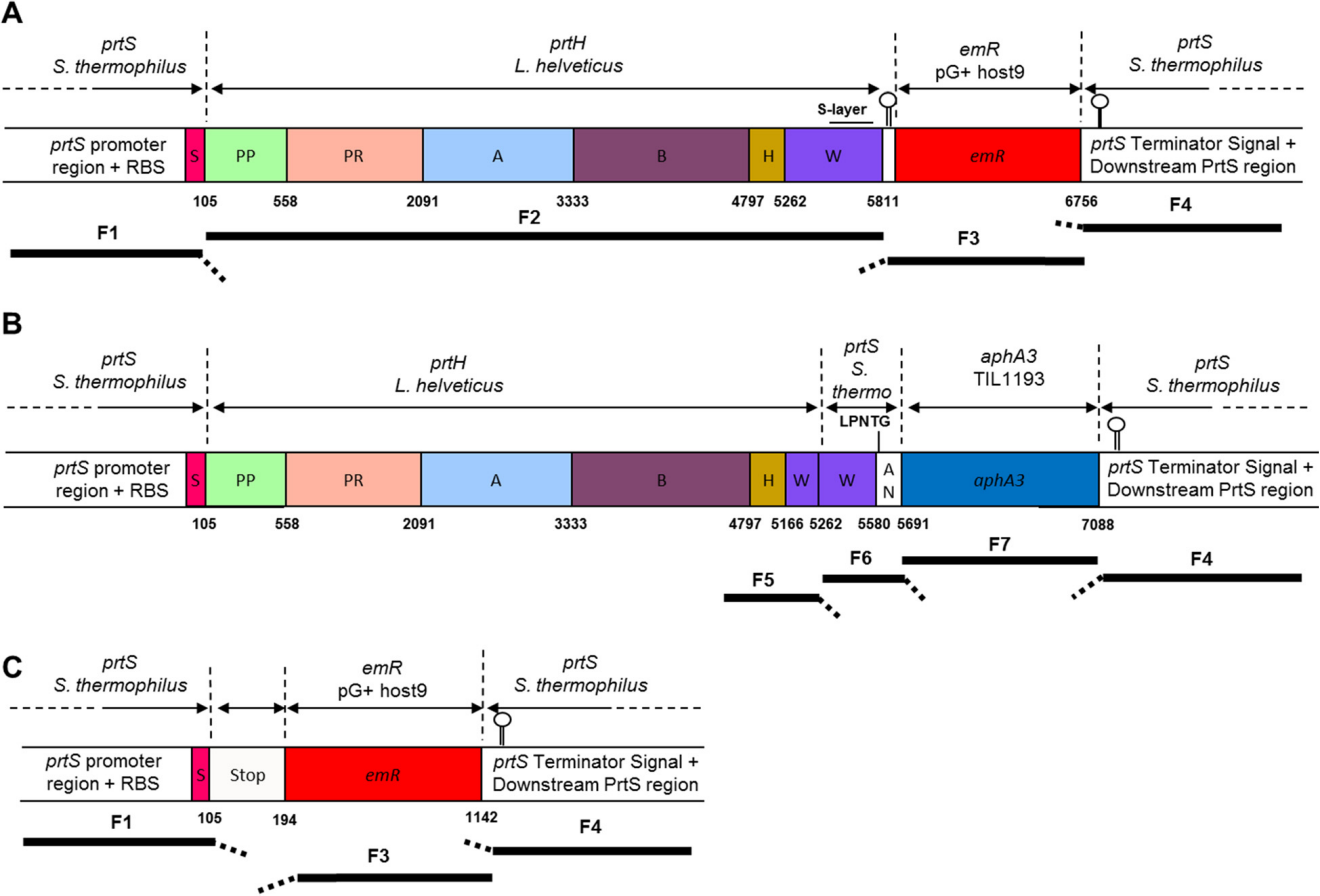
**Schematic representation of the fragments used for mutant constructions and resulting mutant loci.** Mutant constructions were performed by fusing different fragments from *prtS*, *prtH*, *emR* and *aphA3* genes named F1 to F7 (black bars) to produce a PrtH fusion protein. Fragments tails (dashed thick bars) are homologous to the beginning of adjacent fragment in the construction. Numbers in bold characters refer to the last nucleotide of a domain. Nucleotides are numbered starting from the adenosine nucleotide of the start codon of the ORF. **A)** PrtH^+^ construction was inserted in *S. thermophilus* LMD-9 to express *L. helveticus* CNRZ32 CIRM-BIA 103 CEP (PrtH), instead of its own CEP (PrtS). In *prtS* chromosome locus, *prtS* promoter, *prtS* RBS and *prtS* S domain were fused to *prtH* gene composed of PP (propeptide domain), PR (catalytic domain), A (A domain), B (B domain), H (helix domain), W (cell-wall spacer domain) with a 303 nucleotides (nt) S-layer attachment domain (small bar) and a downstream region with the *prtH* terminator signal. An erythromycin resistance gene (*emR*) from pG+host9 [48] was inserted between *prtH* and the downstream region of *prtS*. **B)** PrtH^+^WANS construction replaced the last 450 nt, containing S-layer attachment of *prtH* W domain and *emR* gene from PrtH^+^ mutant, by *prtS* W and AN domains and *aphA3* (kanamycin resistance gene) from *S. thermophilus* TIL1193 strain [18]. The secreted fusion PrtH^+^WANS proteinase has been designed to be covalently anchored to the host peptidoglycan thanks to the PrtS LPNTG motif. The PrtS W domain was expecting to bring the fused proteinase above the cell-wall of *S. thermophilus*. **C)** PrtS^-^ sequence was obtained from a clone selected among PrtH^+^ potential mutants where the F2 fragment (*prtH* ORF) was not inserted. A 89 nt sequence composed by the two F2 primers (with 2 mutations) replaced F2 fragment in the construction.

*prtS* of *S. thermophilus* and *prtH* of *L. helveticus* both encode high molecular weight proteins (above 180 kDa) secreted on the cell-wall and belonging to the subtilisin-like serine proteinase family. They display a similar organization, which can be separated into five regions important for our genetic constructions (Figure 1A and B): i) the promoter and the ribosome binding site (RBS); ii) the export signal with the signal sequence (S) which addresses the protein to the secretory system (Sec system); iii) the different CEP domains: pro-peptide domain (PP), catalytic domain (PR), A domain (A), B domain (B) only for *prtH* and helix domain (H); iv) the CEP fixation part consisting of a cell-wall spacer domain (W) and an anchor domain (AN) with a LPXTG anchoring motif for *prtS*, and of a cell-wall spacer domain (W) with a S-layer domain attachment at the C-terminal end for *prtH*; v) a stop codon at the end of the last domain followed by a transcription terminator [25,26]. Three main parameters should be considered to achieve PrtH secretion and anchoring: the signal sequence, the transcription level of the CEP and the anchoring motif. As *S. thermophilus* LMD-9 expresses an active CEP on its cell-wall, we have chosen to use the expression/secretion pathway of PrtS to express and secrete the CEP of *L. helveticus*. Thus, our strategy to secrete PrtH was to replace the pro-protein sequence of *prtS* of *S. thermophilus* LMD-9 with the pro-protein sequence of *prtH* of *L. helveticus* CNRZ32 (from the PP to the W domains), maintaining the promoter, RBS, and signal sequence of *prtS* (Figure 2A and B).

Regarding the signal sequence, the use of the PRED TAT tool [27] revealed that both CEPs PrtS and PrtH have a signal peptide which addresses them to the Sec system. In gram positive bacteria , this general secretion pathway seems to imply the presence of a peculiar amino acid sequence which follows the signal peptide and could be recognised by the secretory system as previously demonstrated in *Lc. lactis*[2]. As both PrtS and PrtH are predicted to be secreted by the Sec system, this peculiar sequence should be functional in both CEPs. Therefore, we assumed that this peculiar sequence, located at the beginning of the PP domain of PrtH, would be functional in the Sec system of *S. thermophilus*. So only the S domain of PrtS was systematically conserved in our protein constructions to preserve the entire PP domain of PrtH in *S. thermophilus*.

Concerning the anchoring, we decided to test two different cell-wall anchoring systems: either the one from PrtH (S-layer type) or that from PrtS (LPXTG). Indeed, the S-layer motif used by *L. helveticus* may be not adapted to the cell-wall of *S. thermophilus* and PrtH may not correctly interact with the cell-wall. Thus, to maximise the anchoring of the proteinase, we replaced its S-layer domain by the W and AN domains of PrtS.

### Constructions of PrtH^+^, PrtH^+^WANS and PrtS^-^ mutants

In order to heterologously express PrtH on the cell-wall of *S. thermophilus* LMD-9, we constructed two mutants with two different anchoring systems (Figure 2). The mutant constructions were based on the design of an overlapping PCR (OL PCR) fragment to join the different gene parts, the natural transformation of *S. thermophilus* with the OL PCR resulting fragment and its integration into the chromosome by double cross-over recombination. The first mutant, named PrtH^+^, was expected to secrete PrtH which exhibited at the C-terminal of its W domain a S-layer anchoring motif, thought to anchor PrtH to the *L. helveticus* cell-wall [24,28] (Figure 2A). The 8622 base pair (bp) construction (F1 to F4) allowed the insertion of a 6718 bp foreign DNA by double cross-over recombination, replacing a large part of the *prtS* locus in *S. thermophilus* LMD-9 chromosome. As expected, the heterologous fused gene comprised the promoter, the RBS and the Signal sequence (S domain) of *prtS*, the *prtH* ORF spanning from the PP domain to 46 nucleotides (nt) downstream the stop codon, an erythromycin resistance gene and the downstream region of *prtS*.

The second mutant, named PrtH^+^WANS, derived from the first. The anchoring system of the PrtH^+^ mutant was replaced by the *prtS* cell-wall spacer and the anchor domains (W and AN domains) of *S. thermophilus* (Figure 2B and Methods). This was chosen to favour the secretion and the anchoring of the PrtH CEP in the cell-wall of *S. thermophilus* by covalent binding. Thus, a 4186 bp OL PCR fragment (Figure 2B; F5-F6-F7-F4) replaced by double cross-over recombination the *prtH* W domain (from the 97th nt of *prtH* W domain to the end of F3 fragment) by the F6 and F7 fragments.

During the construction of the PrtH^+^ mutant, one of the erythromycin resistant clones was lacking the *prtH* gene fragment (F2) (Figure 2C). In this mutant, called PrtS^-^, the major part of *prtS* was replaced by an 89 nt sequence and an erythromycin resistance gene, inserted between F1 and F4 fragments as described in the Methods section. This 89 nt sequence was produced during the OL PCR amplification of *prtH*^*+*^ fragment by the fusion of F1 and F3 tail fragments. This sequence was composed of the beginning of *prtH* PP domain directly fused to the 3′ region of F2 (after *prtH* stop codon). These two fragments had mutations (data not shown) and the resulting 48 aa residues peptide was composed of *prtS* S domain (MKKKETFSLRKYKIGTVSVLLGAVFLFAGAPSVAA) followed by 13 aa residues (KQQVKASVDSQTK) similar to the N-terminal aa residues of the PrtH PP domain with one mutation changing the first aa residue glutamate (E) of the PP into a lysine residue (K). As PrtS^-^ mutant was deprived of the major part of *prtS* and no other CEP genes were present in *S. thermophilus* LMD-9 genome, this mutant was used as a negative control in this study.

For the three constructed mutants, the constructions were checked by sequencing, which also confirmed that no additional or unexpected mutations occurred in the three mutant sequences.

### Heterologous gene expressions

**Figure 3:**
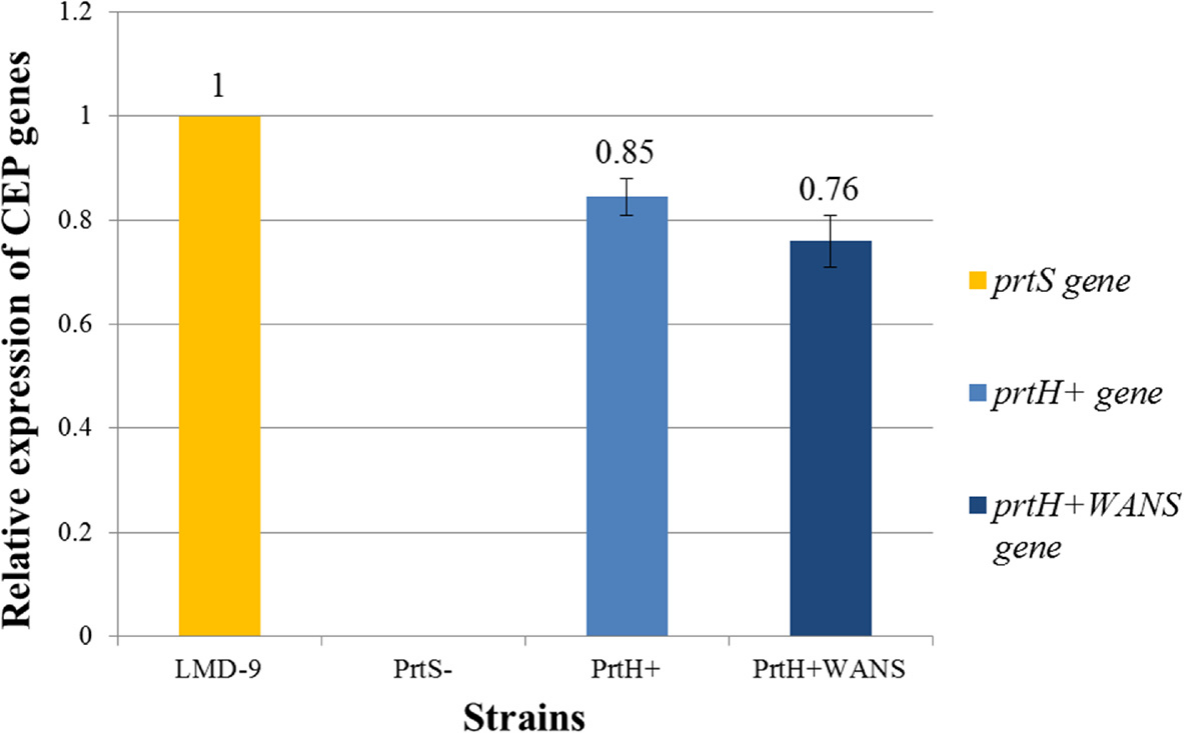
**Relative expression of CEP genes in the mutants compared to *prtS *in *S. thermophilus *LMD-9.** The expression of CEP genes (*prtS*, *prtH*^*+*^ and *prtH*^*+*^*WANS*) was detected by RT-qPCR of *S. thermophilus* wild-type LMD-9 and PrtS^-^, PrtH^+^ and PrtH^+^WANS mutant strains during the exponential growth phase in milk. CEP gene expressions were normalised with respect to the reference gene sigma70/sigma32 for each strain. The normalised expressions of *prtH*^*+*^*and prtH*^*+*^*WANS* CEP genes were compared to that of the reference *prtS* gene.

The transcription of *prtH*^*+*^ and *prtH*^*+*^*WANS* genes was investigated by real-time quantitative polymerisation chain reaction (RT-qPCR) during the exponential growth of *S. thermophilus* PrtH^+^ and PrtH^+^WANS in milk (Figure 3). This medium was use to maximise the CEP gene expression. Indeed, to grow at high cellular density in milk, which is poor in free amino acids and peptides, *S. thermophilus* requires an efficient CEP activity to hydrolyse caseins into peptides for nitrogen supply, and this activity has been shown to be maximum during the exponential growth phase of the bacterium [29]. As expected, the *prtS*, *prtH*^*+*^ and *prtH*^*+*^*WANS* genes were expressed in *S. thermophilus* LMD-9 wild-type, PrtH^+^ mutant and PrtH^+^WANS mutant, respectively. When compared with the expression level of the *prtS* gene in the wild-type strain, the *prtH*^*+*^ and *prtH*^*+*^*WANS* genes appeared to be approximately 15% and 24% lower, respectively. No CEP gene expression was detected in the PrtS^-^ mutant.

Although CEP genes were expressed in PrtH^+^ and PrtH^+^WANS mutants, their growth in skim milk appeared to be very similar to that of the PrtS^-^ mutant. These three mutants grew slightly lower than the wild-type strain LMD-9. Indeed, the three mutants reached an optical density at 480 nm (OD_480 nm_) of 2 in five hours whereas the wild-type strain reached this OD in less than three hours. Additional activity tests were performed by using β- and α_s1_- caseins as substrate as described by Sadat-Mekmene et al. [30]. No CEP activity was detected neither on the cell surface nor in the extracellular culture medium of the PrtH^+^ and PrtH^+^WANS mutants, whereas activity was detected for both wild-type strains *S. thermophilus* LMD-9 and *L. helveticus* CNRZ32 CIRM-BIA 103 (data not shown). However, as the strain CIRM-BIA 103 contains 4 protease genes, the detected activity may be due to one or more proteases and not necessarily to PrtH.

### Detection of the heterologous proteins expressed on the bacterial cell surface

To detect the presence of the various proteinases at the surface of *S. thermophilus* LMD-9 wild-type and mutant strains, a shaving approach was used. This method consists in hydrolysing the cell surface proteins of the living bacteria by trypsin under mild conditions, and in identifying the peptides released from the surface proteins using chromatographic separation coupled on line with tandem mass spectrometry. For each sample, negative controls without trypsin (cf. Methods) were performed. The identified proteins are reported in Additional file 1: shaving results A. The three CEPs PrtS, PrtH^+^ and PrtH^+^WANS were identified on the cell-wall of *S. thermophilus* LMD-9 wild-type strain, PrtH^+^ and PrtH^+^WANS mutants, respectively (Table 1 and Additional file 1: shaving results B, C and D). All detected peptides were specific to trypsin hydrolysis (which cuts after lysyl and arginyl amino acid residues), suggesting that these peptides did not result from degradation by another protease. To estimate the coverage of each protein identified by LC-MS/MS experiments, the first step was to calculate the protein abundance index (PAI) which is the number of observed peptides divided by the number of observable peptides per protein. The second step was to calculate the exponentially modified protein abundance index (emPAI) from the PAI values as: emPAI = 10^PAI^-1. Although shaving is first of all a qualitative method, the emPAI allows a semi quantitative estimation of proteins regarding each sample separately [31,32].

**Table 1:** **Proteinases identified after cell surface shaving of *S. thermophilus *** **strains PrtS**^**-**^**, LMD-9, PrtH**^**+**^**and PrtH**^**+**^**WANS**

			**PrtS**^**-**^		**LMD-9**		**PrtH**^**+**^		**PrtH**^**+**^**WANS**	
**Description**	**Prot Id**	**MW (kDa)**	**Nb id pept**	**emPAI**	**Nb id pept**	**emPAI**	**Nb id pept**	**emPAI**	**Nb id pept**	**emPAI**
Subtilisin-like serine protease (PrtS)	Q03L35	172.9			60	112.92				
PrtH^+^		208.6					40	6.86		
PrtH^+^WANS		206.0							82	161.66

emPAI: exponentially modified Protein Abundance Index [31].

Nb id pept: number of identified peptides belonging to identified protein.

Prot Id: Identification sequence from the following databases TrEMBL.

**Figure 4:**
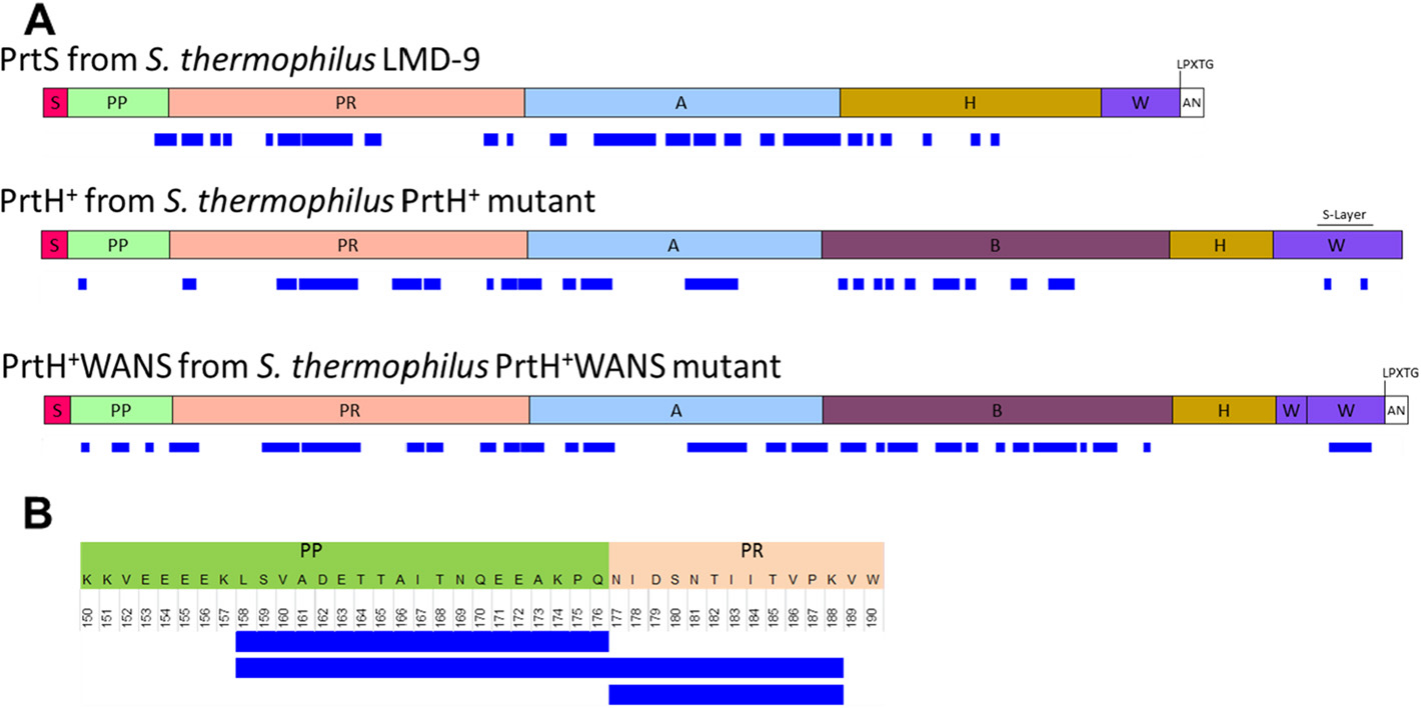
**Schematic location of peptides identified from proteinase sequences after cell surface shaving.** Cell surface proteins of *S. thermophilus* LMD-9, PrtH^+^, PrtH^+^WANS strains were hydrolysed by trypsin and peptides were identified by chromatography coupled on line with ESI quadrupole-orbitrap tandem mass spectrometry. **A**. Peptides corresponding to CEPs were aligned with PrtS, PrtH^+^ and PrtH^+^WANS protein sequences. Peptide sequences are represented with blue bars under the corresponding domains of CEPs: S, PP, PR, A, B, W and AN. **B**. Peptides identified (blue bars) around the cleavage site between PP and PR domains of *S. thermophilus* LMD-9 PrtS CEP. Amino acids and their position from the S domain of PrtS are indicated.

The emPAI of CEPs among identified proteins in the sample was 112.92 for PrtS, 6.86 for PrtH^+^ and 161.66 for PrtH^+^WANS (Table 1). PrtS and PrtH^+^WANS were thus ranked among the most abundant proteins detected in their respective samples (Additional file 1: shaving results A). Those most abundant proteins were also predicted as cell surface proteins. So PrtH^+^WANS seemed to be present at the cell surface, probably anchored to the cell-wall as it is the case for PrtS. In contrast, PrtH^+^ appeared to be less abundant among the identified proteins. Identified CEP peptides are presented in Figure 4 and in Additional file 1: shaving results B, C and D. For PrtS, 60 peptides were identified from the PP to H domains, while no peptides corresponded to the S, W or AN domains (Figure 4A and Additional file 1: shaving results B). For PrtH^+^, 40 peptides were identified from the PP to the W domains except from the H domain, while no peptides corresponded to the S domain (Figure 4A and Additional file 1: shaving results C). Finally, 82 peptides covering the PrtH^+^WANS sequence from the PP to the PrtS W domains were detected while no peptides originating from the S and AN domains of PrtS or from the H and W domains of PrtH were detected (Figure 4A and Additional file 1: shaving results D). As expected, no CEP was identified on the cell surface of the PrtS^-^ mutant.

## Discussion

Although tools required for the expression of heterologous proteins in LAB have been recently developed [4], *B. subtilis* and *E. coli* remain the mostly employed bacteria [1,3]. However, the expression of proteins on the bacterial surface remains a challenge regardless the bacterial host because the final structure of the secreted protein is influenced by various parameters. For example, pH, temperature or ionic environment can impair the final conformation and subsequent function of the heterologous protein. Moreover, the cell-wall composition (peptidoglycan, S-layer, …), which differed from one species to another [33,34], should influence the protein environment. Thus, enlarging the host panel to express heterologous proteins on the cell surface offers new choices between various secretion or cell-wall anchoring systems. LAB host can also be used as vectors in various milk derived products. To the best of our knowledge, this study reports for the first time the secretion to the cell-wall of a high molecular weight heterologous protein (above 200 kDa), i.e. the CEP PrtH of *L. helveticus*. Two mutants were constructed by natural transformation to secrete instead of the CEP PrtS of *S. thermophilus* LMD-9 wild-type strain, a PrtH (with its own S-layer attachment motif) and a PrtH^+^WANS protein (where the PrtH anchoring system was replaced by the PrtS one).

### CEPs identification and location by shaving

To determine whether proteinases were really present on the cell surface, even at low level, we chose to shave the surface of the live cells of *S. thermophilus* wild-type and mutant strains by trypsin, a proteinase of well-known specificity, widely used in proteomics for protein identification by gel or gel-free based methods. This leads to the release of peptides from the cell surface proteins. This study presents, to the best of our knowledge, the first use of the shaving method on a *S. thermophilus* strain. The results have been very successful, not only because the shaving allowed us proving that the heterologous proteins were actually expressed but it also allowed detecting other proteins on the cell surface of the mutants and the wild-type strain. Forty-three proteins were thus identified. Searching their subcellular locations either by using the Uniprot data bank or by similarity to subcellular located proteins [35] revealed 17 proteins defined as cell surface (CS), or membrane-located (M) proteins, 22 cytoplasmic proteins and four uncharacterised proteins (Additional file 1: shaving results A). Only two cytoplasmic proteins were identified in all samples: the elongation factor Tu and the ATP-binding subunit of Clp protease and DnaK/DnaJ chaperones (Additional file 1: shaving results A). To estimate the abundance of each protein in each sample compared to all detected proteins, we calculated the protein content in percent using the following formula: ((emPAI of CS + M)/total emPAI)×100 [31]. Thus extracellular proteins (CS + M) represented 99.97%, 99.92%, 98.82% and 99.92% of all detected proteins for PrtS^-^ mutant, LMD-9, PrtH^+^ mutant and PrtH^+^WANS mutant, respectively.

The identification of few intracellular proteins, at a low level, is intrinsic to shaving [36] and can be explained either by a slight cell lysis during shaving leading to the release of intracellular proteins [37] or to the presence of moonlighting multitask proteins such as enolase [38].

Regarding the CEPs, PrtS, PrtH^+^ and PrtH^+^WANS were identified in shaving supernatants of *S. thermophilus* LMD-9 wild-type strain, PrtH^+^ mutant and PrtH^+^WANS mutant, respectively, while no CEP was identified for PrtS^-^ mutant strain (Table 1). As no signal peptide was identified, this suggests that the three CEPs were excreted through the Sec system, and consequently located on the cell-wall of *S. thermophilus*. So, if the first amino acids following the signal sequence are crucial for the translocation by the sec system (see “Strategy of the mutant constructions” in the Results part), we showed that the peculiar sequence present in PrtH of *L. helveticus* is functional in *S. thermophilus*. The wild-type strain *S. thermophilus* LMD-9 secretes PrtS CEP covalently anchored to the cell-wall [39]. This protein was highly abundant as shown by the high value of the emPAI (Table 1 and Additional file 1: shaving results A). This was also the case for PrtH^+^WANS, but not for PrtH^+^. This suggests that PrtH^+^WANS is probably covalently anchored to the cell-wall of *S. thermophilus* LMD-9, by the action of the sortase SrtA which recognizes the LPXTG motif, as it is the case for PrtS. Thus, the anchored system could be crucial for having highly abundant protein at the cell surface and it appears that the S-layer motif arising from *L. helveticus* strain seemed less adapted than the LPXTG motif to attach proteins to the cell-wall of *S. thermophilus*. Indeed, the emPAI of PrtH^+^ don’t rank it among the most abundant proteins detected in its sample, contrary to PrtH^+^WANS (Additional file 1: shaving results A). This difference is also encountered with the number of identified peptides as 40 peptides were identified for CEP of PrtH^+^ mutant against 84 peptides for PrtH^+^WANS mutant (Figure 4A and Additional file 1: shaving results C and D). The lower number of PrtH^+^ identified peptides could be explained by a lower interaction of PrtH^+^ with the cell-wall leading to its higher release into the extracellular medium. Indeed, the absence of S-layer proteins surrounding the cell of *S. thermophilus* could have impaired the interaction between the W domain of PrtH and the peptidoglycan [33,34]. Actually, Hu et al. [40] showed that an excess of fusion green fluorescent protein (GFP) harbouring a S-layer motif incubated one hour with *S. thermophilus* and *Lc. lactis* cells was able to be fixed on their surface but at low level compared to other LAB expressing S-layer proteins on their cell-wall.

Regarding the PrtS maturation, peptide LSVADETTAITNQEEAKPQ, corresponding to the C-terminal extremity of the PP domain, and peptide NIDSNTIITVPK to the N-terminal end of the PR domain were detected, which does not correspond to the theoretical cleavage sites for trypsin (Figure 4B). They could result from a cleavage between the last residue (Q) of the PP domain and the first residue (N) of the PR domain that occurs during the maturation process of PrtS, as shown by Fernandez-Espla et al. [25] and Chang et al. [41]. The concomitant presence of the complete sequence of the peptide LSVADETTAITNQEEAKPQNIDSNTIITVPK suggested that some PrtS molecules were also in their immature form, as stated by Chang et al. [41]. As both mature and immature forms were present at the cell surface, the maturation step could occur after the CEP anchoring.

### CEP activity issues

The activity of PrtH^+^ and PrtH^+^WANS was neither detected at the cell surface of *S. thermophilus* nor in the medium. Moreover, cell-wall heterologous CEPs secreted in *S. thermophilus* mutants were not active on β and α_s1_- caseins in contrast to the cells of wild-type strains of *L. helveticus*[30] and *S. thermophilus* LMD-9 (results not shown). This lack of activity could explain the difference of growth rate observed in milk between mutants and the wild-type strain. One hypothesis to explain the inactivity of the PrtH is the presence of a mutation in the gene sequence. Indeed, in this study, the strain *L. helveticus* CNRZ32 CIRM-BIA 103 was used (Accession: PRJEB1537; Taxonomy ID: 1226332) and the analysis of its *prtH* gene sequence revealed an unexpected difference compared to that of *L. helveticus* CNRZ32 *prtH* sequence previously published [GenBank: AF133727.1; GI: 5758038] although both strains derived from the same initial strain of the CNRZ collection. This difference consisted of a 83 aa residues imperfect duplication which overlaps the end of the H domain and the beginning of the W domain. This insertion, which extends twice the helix domain H, may change the conformation of the protein, the way it can be included in the cell wall or the environment around the catalytic domain PR. Previous works showed that the strains of *L. helveticus* CIRM-BIA 103 and CNRZ32 contain four protease genes, and showed a similar specific activity of the cell envelope proteinases [30], but it is not possible to know whether all CEPs are really active or only some of them.

A second hypothesis is that the heterologous PrtH could be immature. This was observed for PrtH^+^WANS mutant, with the identification of the peptide VYYANDSSADNMANVSTVWNNYK (Additional file 1: shaving results D) which overlaps the PP and PR domains of PrtH^+^WANS CEP. In the case of *L. helveticus*, the maturation process could involve another actor to obtain a functional CEP. *L. helveticus* CNRZ32 has two maturation proteins in the chromosome belonging to the peptidylprolyl cis/trans isomerase family [23], named PrtM [UniProtKB/TrEMBL: A4UAD9] and PrtM2 [UniProtKB/TrEMBL: A4UAE0]. According to Broadbent et al*.*[23], PrtM could be required for PrtH maturation whereas PrtM2 should have a role in maturation of the other *L. helveticus* CEPs. Even if the maturation mechanism on *S. thermophilus* cell-wall is still not well established, this species does not seem to secrete maturase [5]. Maturation of PrtS could be achieved through an automaturation process or other factors not yet identified. One potential experiment to promote maturation of PrtH on the cell surface could be to introduce the *prtM* gene from *L. helveticus* CNRZ32 in PrtH^+^WANS mutant chromosome. However, this experiment requires multiple mutant constructions to expect to obtain active heterologous proteinases. Indeed, the expression level required to efficiently act on PrtH and the location and anchoring of PrtM on the bacterium surface are still ambiguous.

Finally, the lack of activity could be due to the chosen host and/or to the selected heterologous protein. Indeed, we cannot exclude that the microenvironment of the cell-wall (presence or absence of S-layer proteins) may influence the correct folding of the enzyme. This latter problem is also encountered in the other hosts commonly used for heterologous secretion. Furthermore, although PrtS activity on the cell-wall of *S. thermophilus* was demonstrated [29,41,42], the quantity of secreted proteins by this pathway is still unknown in *S. thermophilus* LMD-9 and heterologous CEPs as well as PrtS may be displayed at a too low level on the cell surface to detect the activity of heterologous CEPs. Anyway, despite the lack of activity, our tool actually allows secretion and anchoring of proteins of high molecular weight at the cell surface of *S. thermophilus*.

## Conclusion

This study demonstrated that *S. thermophilus* LMD-9 strain is able to secrete and probably anchor heterologous proteins on its cell-wall and gives a new tool to enlarge the panel of heterologous secretion hosts. This *S. thermophilus* strain is a powerful tool regarding its ability for natural competence that allows bypassing plasmid construction to produce recombinant proteins. Moreover, its food grade status added to its wide industrial use and its ability to survive in gastrointestinal tract make *S. thermophilus* LMD-9 a good candidate to release recombinant proteins in a dairy matrix or directly in gastrointestinal tract. At this stage, our tool requires selection genes to be constructed which could constitute a negative point in most applications. However, we can imagine to dispense with the use of a selection gene for further constructions, thanks to the highly transformation rate of *S. thermophilus* LMD-9 [14,15,17].

## Methods

### Bacterial strains and growth conditions

Strains used in this work were *Lactobacillus helveticus* CNRZ32 CIRM-BIA 103, *Streptococcus thermophilus* LMD-9 [43], *S. thermophilus* LMD-9 TIL1193 [18] and three *S. thermophilus* LMD-9 mutants: PrtH^+^, PrtS^-^ and PrtH^+^WANS (this study). They were cultivated at 42°C, in MRS (Difco) for *L. helveticus* and in M17 supplemented with 2% of lactose (LM17) [44] for *S. thermophilus* strains. For natural transformation experiments, LM17 and reconstituted chemically defined medium (CDM) supplemented with 2% of lactose (LCDM) [45] were used. A 10% reconstituted skim milk was used for strain conservation and precultures of *S. thermophilus* strains. When necessary, antibiotics were added to the media: erythromycin (Sigma) at 5 μg.mL^-1^ (for cultures of PrtH^+^ and PrtS^-^ mutants) or kanamycin (Sigma) at 1 mg.mL^-1^ (for culture of PrtH^+^WANS mutant). The absorbance at 480 nm of skim milk cultures was measured after 1:10 dilution in a clarification solution of EDTA 0.2% (pH 12).

### DNA extraction and PCR

General molecular biology techniques, DNA extraction and PCR amplifications were achieved according to Green and Sambrook [46], and/or supplier’s recommendations. PCR fragments were obtained using the Taq DNA polymerase or the Phusion high fidelity DNA polymerase (Fermentas, Saint Rémy-lès-Chevreuse, France) in a Mastercycler proS (Eppendorf). All primers used in this work are listed in Table 2 and in Additional file 2: sequencing primers and Additional file 3: qPCR primers. They were designed by using Primer3Plus [47] and purchased from Eurogentec (Seraing, Belgium). To produce overlapping regions between adjacent fragments (see above), a short nucleotide sequence homologous to the beginning of the adjacent fragment was added at the 5′ end of one of the primer used to amplify the desired fragment (in italics in Table 2).

**Table 2:** **Primers and resulting fragments used in overlap extension PCR for construction of PrtH**^**+**^**and PrtH**^**+**^**WANS mutants**

**Fragment**	**Primer**	**Sequence (5′ – 3′)**	**Length (bp)**	**DNA matrix (strain)**	**Product size (kbp)**	**Hyb temp (°C)**	**Overlap product size (kbp)**	**Overlap hyb temp (°C)**
PrtH^+^								
F1	Style 1-1*	ACAAATTCATGCCGTTCATAAG	22	gDNA (LMD-9)	0.986	59.5	8.622	55
	PrtSssUp(H)_R	*GCCTTAACTTGTTGTTC*TGCAGCTACCGATGGTGC	35					
F2	PrtHpp_F	GAACAACAAGTTAAGGCTAGTGTTGACAGCCAAACAAAAAC	41	gDNA (CNRZ32 CIRM-BIA 103)	5.752	62		
	PrtHterm_R	AAAAGAGTAATGATCCTTCTCATTACTCTTTCATTATATGTAAATGATTATTTTAC	56					
F3	EmpG9(H)_F	*GAGAAGGATCATTACTCTTTT*CAAACTTAAGAGTGTGTTGATAGTGC	47	pG+host 9	0.969	59.5		
	EmpG9_R	GGACCTCTTTAGCTCCTTGG	20					
F4	PrtSDn(Em)_F	*CCAAGGAGCTAAAGAGGTCC*ATAATAAAACCGCTTAATCATTGTG	45	gDNA (LMD-9)	0.973	49		
	PrtSDn_R	CGTCTATCAATCTTGTATTTTCTTG	25					
PrtH^+^WANS								
F5	UpH_F	CGGTATCAAGTGGGGTACTCG	21	gDNA (PrtH+)	1.395	69	4.186	55
	UpH(WANS)_R	*CTTGCTTGGCTTGCAGA*AACAGGAGCTGCAACTTGGTTATC	41					
F6	WANS_F	TCTGCAAGCCAAGCAAG	17	gDNA (LMD-9)	0.473	58		
	WANS(aphA3)_R	*CTCAAATGGTTCGCTGG*TTAGCAAACTTGTGATAAAGC	38					
F7	aphA3_F**	CCAGCGAACCATTTGAG	17	gDNA (TIL1193)	1.4	54		
	aphA3_R**	GTTGCGGATGTACTTCAG	18					
F4	PrtSDn(aphA3)_F	*CTGAAGTACATCCGCAAC*ATAATAAAACCGCTTAATCATTGTG	43	gDNA (LMD-9)	0.971	61.7		
	PrtSDn_R	CGTCTATCAATCTTGTATTTTCTTG	25					

Primers Style 1-1, PrtSDn_R, and UpH_F correspond to those used for overlapping PCR reaction and italicised nucleotides correspond to homologous tails.

The name, sequence, length, DNA matrix, product size (including the added tails), hybridization temperature (hyb temp), overlap product size and overlap hybridisation temperature (hyb temp) are indicated.

bp (base pairs); kbp (kilobase pairs) and gDNA (genomic DNA) * [42], ** [18].

For DNA constructions, each PCR fragment was first amplified with Phusion high fidelity DNA polymerase with the minimum of DNA matrix i.e. enough to have 500 to 1000 matrix copy per PCR reaction. PCR were performed according to polymerase supplier’s recommendations. Fragments were then purified using high pure PCR product purification kit (Roche Applied Science, Meylan, France) and eluted in elution buffer as recommended by Fontaine et al. [17]. For both constructions produced by OL PCR, the four purified fragments were pooled in equal amount and PCR-amplified, in a final volume of 20 μL, with the forward primer of the 5′ end fragment and the reverse primer of the 3′ end fragment (Table 2). For sequencing, PCR fragments were amplified with the Taq polymerase and sequencing primers (Additional file 2: sequencing primers) and shipped to the company Beckman Coulter Genomics (Essex, U.K.).

### Genetic material and mutant constructions

Four DNA matrices were used for mutant constructions: the *prtH* gene [locus_tag: LHCIRMBIA103_00753; NCBI Accession Number: PRJEB1537; Taxonomy ID: 1226332] of *L. helveticus* CNRZ32 CIRM-BIA 103 (Figure 1A), the *prtS* gene [locus tag: STER_0846; NCBI Reference Sequence: YP_820283.1; GI: 116627664] from *S. thermophilus* LMD-9 (Figure 1B), the erythromycin resistance gene (Figure 1C) from pG+host9 plasmid DNA [48] and the kanamycin resistance gene (Figure 1D) from *S. thermophilus* LMD-9 TIL1193 genomic DNA (gDNA) [18].

Two constructions were obtained using OL PCR. Primers used for OL PCR are described in Table 2. The *prtH*^*+*^ 8622 bp construction was produced by assembling 4 fragments in this order: F1, F2, F3 and F4 (Figure 2). For F1 and F4 respectively, a 969 bp sequence composed of the promoter, RBS, and S domain of *prtS* and the 953 bp downstream region of *prtS* fragment starting 33 nt after *prtS* stop codon were amplified from *S. thermophilus* LMD-9 gDNA (Figure 1B). For F2, a 5752 bp region of the *prtH* open reading frame (ORF) sequence, from PP to 46 nt after stop codon was amplified from *L. helveticus* CNRZ32 CIRM-BIA 103 gDNA (Figure 1A). For F3, a 948 bp sequence (Figure 1C) containing an erythromycin resistance gene was amplified from pG+host9 plasmid DNA [48].

The *prtH*^*+*^*WANS* 4186 bp construction results from the assembly of 4 fragments F5, F6, F7 and F4 (Figure 2B). For F5, a 1378 bp region composed of the last 817 nt of B domain, the H domain and the first 96 nt of W domain of *prtH* excluding the S-layer sequence was amplified from *L. helveticus* CNRZ32 CIRM-BIA 103 gDNA (Figure 1A). For F6, a 456 bp sequence with W and AN domains of *prtS* plus 30 nt after *prtS* stop codon was amplified from *S. thermophilus* LMD-9 gDNA (Figure 1B). For F7, a 1400 bp sequence of kanamycin resistance gene (Figure 1D) was amplified from *S. thermophilus* LMD-9 TIL1193 gDNA [18]. The F4 region was the same as the one used for PrtH^+^ construction.

### Natural transformation

Linear DNA fragments obtained by OL PCR were introduced in *S. thermophilus* LMD-9 by natural transformation as described by Gardan et al*.*[18]. Briefly, *S. thermophilus* LMD-9 was grown at 42°C in chemically defined medium (CDM) during 5–6 hours, until OD_600 nm_ = 1.8-2 (exponential growth phase). Culture was then diluted in CDM in order to obtain OD_600 nm_ = 0.05 and incubated at 42°C about 1 hour, until OD_600 nm_ reached 0.2. Then, 100 μL of culture was mixed with 3 μL of OL PCR product and incubated 30 min at 42°C. After the adding of 900 μL of LM17, the mixture was re-incubated 40 min at 42°C. Cells were then concentrated 10 times by centrifugation (5 min, 3900 g) and resuspension with their own supernatant, and spread on LM17 agar plates supplemented with the appropriate antibiotic.

The *prtH*^*+*^ OL PCR fragment was introduced into *S. thermophilus* LMD-9 to obtain a PrtH^+^ mutant. Likewise, the *prtH*^*+*^*WANS* OL PCR fragment was introduced in the PrtH^+^ mutant to obtain the PrtH^+^WANS mutant. Integration of OL PCR fragments at the targeted locus is achieved through the presence of 2 fragments of approximately 1000 bp homologous to the targeted locus and surrounding the foreign DNA. These homologous regions were included into the F1 or F5, and F4 fragments (Figures 1 and 2).

### RNA extraction, reverse transcription and quantitative real-time PCR

*S. thermophilus* LMD-9, PrtS^-^, PrtH^+^ and PrtH^+^WANS were grown in skim milk up-to OD_480 nm_ = 1–2 (exponential growth phase), before milk coagulation. Milk caseins were removed according Chopard et al. [49]: at 6 mL of skim milk culture were added 795 μL of saline solution (NaCl 0.85% (wt/v); sodium glycerophosphate 0.5% (wt/v); tween 80 0.1% (v/v); pH 7) and 195 μL of trisodium citrate solution 1 M. The cell pellets were harvested by centrifugation (15 min, 3900 g at 4°C) and a second washing with the same solutions was done. The following step was performed using the RNA extract Kit Aurum (Bio-Rad, Marnes La Coquette, France) and cell lysis step was modified as follows: to improve cell lysis, an ultrasonic bath (Bioruptor, Diagenode, Liège, Belgium) (15 s 4 times at maximal power) was performed twice. An additional mechanic milling with 250 mg of Ø 0.1 – 0.25 mm glass beads (Fisher Scientific, Illkirch, France) was also performed in 2 mL microtubes by vortexing first 60 s then twice 30 s, with at least 2 min stay on ice between each milling. The supernatant was collected after centrifugation at 12,000 *g* for 1.5 min at room temperature.

After nucleic acids extraction, two DNAse steps were performed: a first step with DNAse I (Bio-Rad) followed by a phenol-chloroform extraction [46] and a second step with RNase-free DNase I (Ambion, Courtaboeuf, France). The last step was repeated until PCR control with sigma70 primers was negative after 30 PCR cycles [42]. Total RNA concentrations were determined by measuring the absorbance at 260 nm using a spectrophotometer Nanodrop-1000 (Thermo Scientific, Illkirch, France). Complementary DNAs (cDNA) were synthesized from 1 μg of RNA by using Moloney murine leukemia virus reverse transcriptase (Invitrogen, Saint Aubin, France) according to the manufacturer’s instructions.

Quantitative PCR (qPCR) reactions were performed on CFX96 Touch™ Real-Time PCR Detection System (Biorad) following manufacturer’s instructions. qPCR primers were designed with Primer3Plus software [47] to amplify approximately 130 bp fragments of: *sigma70/sigma32* (reference gene), *prtS* (positive control) and *prtH* (Additional file 3: qPCR primers). Serial dilutions of each cDNA sample (LMD-9, PrtH^+^ and PrtH^+^WANS) were carried out to check efficiency of each primer pair and obtain standard curves (Ct = f(log initial cDNA concentration)). Relative quantities were obtained after comparing to the standard curves and normalized using the following formula: R = (relative quantity of gene of interest)/(relative quantity of reference gene). Ratios obtained for *prtH* gene in PrtH^+^ and PrtH^+^WANS mutants were then compared to that of *prtS* gene in LMD-9. Quantification was carried out twice independently and the mean and SEM (Standard Error of Mean) were determined.

### Surface tryptic digestion of live cells (shaving)

*S. thermophilus* LMD-9 wild-type and the three mutants were grown at 42°C in 50 mL of 10% reconstituted skim milk to OD_480 nm_ = 2 (exponential growth phase). Cells were washed first in tri-sodium citrate solution 0.25 M (Carlo Erba, Val de Reuil, France) and centrifuged at 500 *g*, 10 min at 4°C. The supernatants containing bacterial cells were then centrifuged at 8,000 *g*, 10 min at 4°C and washed at least three times with 100 mM Tris–HCl, pH 7.5 containing 150 mM NaCl to remove the residual caseins. Washed cells were concentrated to OD_650 nm_ = 30 and suspended in PBS containing 5 mM DL-Dithiothreitol (Sigma-Aldrich, St Quentin Fallavier, France) solution, adjusted to pH 8.5. Five μg of sequenced grade trypsin (Promega, Charbonnières, France) were added to 500 μL of the concentrated cell solution. In parallel, negative controls were carried out by adding the trypsin buffer without the enzyme. Cells were incubated 1 hour at 37°C with shaking (180 rpm). Supernatants were harvested by centrifugation at 10,000 *g*, 10 min at room temperature and filtrated through a 0.45 μm filter (Millex PVDF, 13 mm, Millipore, Molsheim, France). Supernatants were incubated overnight at 37°C with shaking (100 rpm) in presence of 1 μg of trypsin. The reaction was stopped by adding 15 μL TriFluoroacetic Acid (TFA) 10% (v/v) (Sigma Aldrich) and samples were stored at -20°C before mass spectrometry analysis.

### Tandem mass spectrometry

Mass spectrometry (MS) experiments were performed using a nanoRSLC Dionex U3000 system fitted to a Q Exactive mass spectrometer (Thermo Scientific, San Jose, USA) equipped with a nanoelectrospray ion source. A preliminary sample concentration step was performed on a nanotrap PepMap 100 (C18, 3 μm, 75 μm Inner Diameter (ID) × 20 mm Length (L)) (Dionex, Amsterdam, Netherlands). Separation was performed on a reverse-phase column PepMap RSLC C18 3 μm, 100 Å (75 μm ID, 150 mm L) (Dionex, Amsterdam, Netherlands) at 35°C, using solvent A (2% (v/v) acetonitrile, 0.08% (v/v) formic acid and 0.01% (v/v) TFA in deionized water) and solvent B (95% (v/v) acetonitrile, 0.08% (v/v) formic acid and 0.01% (v/v) TFA in deionized water). 5-60% of solvent B in 46 min and 60-80% in 1 min was applied as separation gradient at a flow rate of 0.3 μL/min. Eluted peptides were directly electrosprayed into the mass spectrometer operated in positive mode and a voltage of 2 kV with the help of a Proxeon Nanospray Flex ion source (Thermo Scientific, San Jose, USA). Spectra were recorded in full MS mode and selected in a mass range 300–2000 *m/z* for MS spectra with a resolution of 70,000 at m/z 200. For each scan, the ten more intense ions were selected for fragmentation. MS/MS spectra were recorded with a resolution of 17,500 at m/z 200 and the parent ion was subsequently excluded of the analysis during 15 s. The instrument was externally calibrated according to the supplier’s procedure.

To identify peptides, all data (MS and MS/MS) were submitted to X! Tandem using the X! Tandem pipeline developed by PAPPSO (Plateforme d’Analyse Protéomique de Paris Sud-Ouest (PAPPSO), INRA, Jouy-en-Josas, France, http://pappso.inra.fr).

The search was performed against a database composed of the taxonomy Bacilli from http://www.uniprot.org (Taxon identifier: 91061) to which was added the deduced sequences of the two proteins of PrtH^+^ and PrtH^+^WANS. Database search parameters were specified as follows: trypsin cleavage was used and the peptide mass tolerance was set to 10 ppm for MS and 0.02 Da for MS/MS. Oxidation of methionine was selected as a variable modification. Semi-tryptic peptides were allowed during the “refinement” process of X!tandem. For each peptide identified, a minimum score corresponding to an e-value below 0.05 was considered as a prerequisite for peptide validation.

The identified proteins were conserved when at least three specific peptides were identified.

## Competing interests

The authors declare that they have no competing interests.

## Authors’ contributions

XL carried out the experiments and contributed to the redaction of the manuscript. VG supervised shaving experiments and results analysis and contributed to the redaction. VBB provided technical assistance for mass spectrometry. JJ provided experience and knowledge for mass spectrometry. SL initiated this work and corrected the manuscript. AD initiated and supervised this work. MG supervised the project and contributed to the redaction of the manuscript. All authors read and approved the final manuscript.

## Supplementary Material

Additional file 1 **Proteins identified after cell surface shaving of *S. thermophilus *** **strains PrtS**^**-**^**, LMD-9, PrtH**^**+**^** and PrtH**^**+**^**WANS.** Click here for file

Additional file 2 Sequencing primers. Click here for file

Additional file 3 Primers used for qPCR experiments. Click here for file

## References

[B1] Bernaudat F Frelet-Barrand A Pochon N Dementin S Hivin P Boutigny S Rioux JB Salvi D Seigneurin-Berny D Richaud P Joyard J Pignol D Sabaty M Desnos T Pebay-Peyroula E Darrouzet E Vernet T Rolland N Heterologous expression of membrane proteins: choosing the appropriate host PLoS One 2011 6 12 e29191 22216205 10.1371/journal.pone.0029191 PMC3244453

[B2] Le Loir Y Azevedo V Oliveira SC Freitas DA Miyoshi A Bermudez-Humaran LG Nouaille S Ribeiro LA Leclercq S Gabriel JE Guimaraes VD Oliveira MN Charlier C Gautier M Langella P Protein secretion in *Lactococcus lactis*: an efficient way to increase the overall heterologous protein production Microb Cell Fact 2005 4 2 15631634 10.1186/1475-2859-4-2 PMC545053

[B3] Westers L Westers H Quax WJ *Bacillus subtilis* as cell factory for pharmaceutical proteins: a biotechnological approach to optimize the host organism Biochim Biophys Acta-Mol Cell Res 2004 1694 299 310 10.1016/j.bbamcr.2004.02.011 15546673

[B4] Morello E Bermudez-Humaran LG Llull D Sole V Miraglio N Langella P Poquet I *Lactococcus lactis*, an efficient cell factory for recombinant protein production and secretion J Mol Microbiol Biotechnol 2008 14 48 58 17957110 10.1159/000106082

[B5] Zhou MM Theunissen D Wels M Siezen RJ LAB-Secretome: a genome-scale comparative analysis of the predicted extracellular and surface-associated proteins of Lactic Acid Bacteria BMC Genomics 2010 11 651 21092245 10.1186/1471-2164-11-651 PMC3017865

[B6] Wydau S Dervyn R Anba J Ehrlich SD Maguin E Conservation of key elements of natural competence in *Lactococcus lactis* ssp FEMS Microbiol Lett 2006 257 32 42 16553829 10.1111/j.1574-6968.2006.00141.x

[B7] Johnsborg O Eldholm V Havarstein LS Natural genetic transformation: prevalence, mechanisms and function Res Microbiol 2007 158 767 778 17997281 10.1016/j.resmic.2007.09.004

[B8] Elli M Callegari ML Ferrari S Bessi E Cattivelli D Soldi S Morelli L Feuillerat NG Antoine JM Survival of yogurt bacteria in the human gut Appl Environ Microbiol 2006 72 5113 5117 16820518 10.1128/AEM.02950-05 PMC1489325

[B9] Mater DDG Bretigny L Firmesse O Flores MJ Mogenet A Bresson JL Corthier G *Streptococcus thermophilus* and *Lactobacillus delbrueckii* subsp. *bulgaricus* survive gastrointestinal transit of healthy volunteers consuming yogurt FEMS Microbiol Lett 2005 250 185 187 16099606 10.1016/j.femsle.2005.07.006

[B10] Guarner F Perdigon G Corthier G Salminen S Koletzko B Morelli L Should yoghurt cultures be considered probiotic? Br J Nutr 2005 93 783 786 16022746 10.1079/bjn20051428

[B11] Solaiman DKY Somkuti GA Construction of a green-fluorescent protein-based, insertion-inactivation shuttle vector for lactic acid bacteria and *Escherichia coli* Biotechnol Lett 1997 19 1175 1179

[B12] Somkuti GA Solaiman DKY Johnson TL Steinberg DH Transfer and expression of A streptomyces cholesterol oxidase gene in *Streptococcus thermophilus* Biotechnol Appl Biochem 1991 13 238 245 2043280

[B13] Renye JA Somkuti GA Vector-mediated chromosomal integration of the glutamate decarboxylase gene in *Streptococcus thermophilus* Biotechnol Lett 2012 34 549 555 22105555 10.1007/s10529-011-0802-6

[B14] Blomqvist T Steinmoen H Havarstein LS Natural genetic transformation: a novel tool for efficient genetic engineering of the dairy bacterium *Streptococcus thermophilus* Appl Environ Microbiol 2006 72 6751 6756 17021227 10.1128/AEM.01156-06 PMC1610297

[B15] Fontaine L Dandoy D Boutry C Delplace B De Frahan MH Fremaux C Horvath P Boyaval P Hols P Development of a versatile procedure based on natural transformation for marker-free targeted genetic modification in *Streptococcus thermophilus* Appl Environ Microbiol 2010 76 7870 7877 20935129 10.1128/AEM.01671-10 PMC2988589

[B16] Fontaine L Goffin P Dubout H Delplace B Baulard A Lecat-Guillet N Chambellon E Gardan R Hols P Mechanism of competence activation by the ComRS signalling system in streptococci Mol Microbiol 2013 87 1113 1132 23323845 10.1111/mmi.12157

[B17] Fontaine L Boutry C De Frahan MH Delplace B Fremaux C Horvath P Boyaval P Hols P A novel pheromone quorum-sensing system controls the development of natural competence in *Streptococcus thermophilus* and *Streptococcus salivarius* J Bacteriol 2010 192 1444 1454 20023010 10.1128/JB.01251-09 PMC2820839

[B18] Gardan R Besset C Guillot A Gitton C Monnet V The oligopeptide transport system is essential for the development of natural competence in *Streptococcus thermophilus* Strain LMD-9 J Bacteriol 2009 191 4647 4655 19447907 10.1128/JB.00257-09 PMC2704715

[B19] Goh YJ Goin C O’Flaherty S Altermann E Hutkins R Specialized adaptation of a lactic acid bacterium to the milk environment: the comparative genomics of *Streptococcus thermophilus* LMD-9 Microb Cell Fact 2011 10 Suppl 1 S22 21995282 10.1186/1475-2859-10-S1-S22 PMC3231929

[B20] Qin JJ Li RQ Raes J Arumugam M Burgdorf KS Manichanh C Nielsen T Pons N Levenez F Yamada T Mende DR Li JH Xu JM Li SC Li DF Cao JJ Wang B Liang HQ Zheng HS Xie YL Tap J Lepage P Bertalan M Batto JM Hansen T Le Paslier D Linneberg A Nielsen HB Pelletier E Renault P A human gut microbial gene catalogue established by metagenomic sequencing Nature 2010 464 U59 U70 10.1038/nature08821 PMC3779803 20203603

[B21] Dandoy D Fremaux C De Frahan MH Horvath P Boyaval P Hols P Fontaine L The fast milk acidifying phenotype of *Streptococcus thermophilus* can be acquired by natural transformation of the genomic island encoding the cell-envelope proteinase PrtS Microb Cell Fact 2011 10 Suppl 1 S21 21995822 10.1186/1475-2859-10-S1-S21 PMC3231928

[B22] Sadat-Mekmene L Genay M Atlan D Lortal S Gagnaire V Original features of cell-envelope proteinases of *Lactobacillus helveticus.* A review Int J Food Microbiol 2011 146 1 13 21354644 10.1016/j.ijfoodmicro.2011.01.039

[B23] Broadbent JR Cai H Larsen RL Hughes JE Welker DL De Carvalho VG Tompkins TA Ardo Y Vogensen F De Lorentiis A Gatti M Neviani E Steele JL Genetic diversity in proteolytic enzymes and amino acid metabolism among *Lactobacillus helveticus* strains J Dairy Sci 2011 94 4313 4328 21854904 10.3168/jds.2010-4068

[B24] Genay M Sadat L Gagnaire V Lortal S *prtH2,* not *prtH*, is the ubiquitous cell-wall proteinase gene in *Lactobacillus helveticus* Appl Environ Microbiol 2009 75 3238 3249 19286786 10.1128/AEM.02395-08 PMC2681653

[B25] Fernandez-Espla MD Garault P Monnet V Rul F *Streptococcus thermophilus* cell wall-anchored proteinase: release, purification and biochemical and genetic characterization Appl Environ Microbiol 2000 66 4772 4778 11055922 10.1128/aem.66.11.4772-4778.2000 PMC92378

[B26] Siezen RJ Multi-domain, cell-envelope proteinases of lactic acid bacteria Antonie Van Leeuwenhoek 1999 76 139 155 10532376

[B27] Bagos PG Nikolaou EP Liakopoulos TD Tsirigos KD Combined prediction of Tat and Sec signal peptides with hidden Markov models Bioinformatics 2010 26 2811 2817 20847219 10.1093/bioinformatics/btq530

[B28] Pederson JA Mileski GJ Weimer BC Steele JL Genetic characterization of a cell envelope-associated proteinase from *Lactobacillus helveticus* CNRZ32 J Bacteriol 1999 181 4592 4597 10419958 10.1128/jb.181.15.4592-4597.1999 PMC103591

[B29] Shahbal S Hemme D Renault P Characterization of a cell envelope-associated proteinase activity from *Streptococcus thermophilus* H-strains Appl Environ Microbiol 1993 59 177 182 16348841 10.1128/aem.59.1.177-182.1993 PMC202074

[B30] Sadat-Mekmene L Jardin J Corre C Mollé D Richoux R Delage MM Lortal S Gagnaire V Simultaneous presence of PrtH and PrtH2 proteinases in *Lactobacillus helveticus* Strains improves breakdown of the pure alphas1-casein Appl Environ Microbiol 2011 77 179 186 21037305 10.1128/AEM.01466-10 PMC3019737

[B31] Ishihama Y Oda Y Tabata T Sato T Nagasu T Rappsilber J Mann M Exponentially modified protein abundance index (emPAI) for estimation of absolute protein amount in proteomics by the number of sequenced peptides per protein Mol Cell Proteomics 2005 4 1265 1272 15958392 10.1074/mcp.M500061-MCP200

[B32] Rappsilber J Ryder U Lamond AI Mann M Large-scale proteomic analysis of the human splicesome Genome Res 2002 12 1231 1245 12176931 10.1101/gr.473902 PMC186633

[B33] Delcour J Ferain T Deghorain M Palumbo E Hols P The biosynthesis and functionality of the cell-wall of lactic acid bacteria Antonie Van Leeuwenhoek 1999 76 159 184 10532377

[B34] Schleifer KH Kandler O Peptidoglycan types of bacterial cell walls and their taxonomic implications Bacteriol Rev 1972 36 407 477 4568761 10.1128/br.36.4.407-477.1972 PMC408328

[B35] Camacho C Coulouris G Avagyan V Ma N Papadopoulos J Bealer K Madden TL BLAST plus: architecture and applications BMC Bioinforma 2009 10 421 10.1186/1471-2105-10-421 PMC2803857 20003500

[B36] Solis N Larsen MR Cordwell SJ Improved accuracy of cell surface shaving proteomics in *Staphylococcus aureus* using a false-positive control Proteomics 2010 10 2037 2049 20217865 10.1002/pmic.200900564

[B37] Olaya-Abril A Gomez-Gascon L Jimenez-Munguia I Obando I Rodriguez-Ortega MJ Another turn of the screw in shaving Gram-positive bacteria: Optimization of proteomics surface protein identification in *Streptococcus pneumoniae* J Proteomics 2012 75 3733 3746 22575384 10.1016/j.jprot.2012.04.037

[B38] Henderson B Martin A Bacterial virulence in the moonlight: Multitasking bacterial moonlighting proteins are virulence determinants in infectious disease Infect Immun 2011 79 3476 3491 21646455 10.1128/IAI.00179-11 PMC3165470

[B39] Marraffini LA Dedent AC Schneewind O Sortases and the art of anchoring proteins to the envelopes of gram-positive bacteria Microbiol Mol Biol Rev 2006 70 192 221 16524923 10.1128/MMBR.70.1.192-221.2006 PMC1393253

[B40] Hu SM Kong J Sun ZL Han LL Kong WT Yang P Heterologous protein display on the cell surface of lactic acid bacteria mediated by the s-layer protein Microb Cell Fact 2011 10 86 22035337 10.1186/1475-2859-10-86 PMC3215925

[B41] Chang OK Perrin C Galia W Saulnier F Miclo L Roux E Driou A Humbert G Dary A Release of the cell-envelope protease PrtS in the growth medium of *Streptococcus thermophilus* 4 F44 Int Dairy J 2012 23 91 98

[B42] Galia W Perrin C Genay M Dary A Variability and molecular typing of *Streptococcus thermophilus* strains displaying different proteolytic and acidifying properties Int Dairy J 2009 19 89 95

[B43] Makarova K Slesarev A Wolf Y Sorokin A Mirkin B Koonin E Pavlov A Pavlova N Karamychev V Polouchine N Shakhova V Grigoriev I Lou Y Rohksar D Lucas S Huang K Goodstein DM Hawkins T Plengvidhya V Welker D Hughes J Goh Y Benson A Baldwin K Lee JH Diaz-Muniz I Dosti B Smeianov V Wechter W Barabote R Comparative genomics of the lactic acid bacteria Proc Natl Acad Sci U S A 2006 103 15611 15616 17030793 10.1073/pnas.0607117103 PMC1622870

[B44] Terzaghi BE Sandine WE Improved medium for lactic Streptococci and their bacteriophages Appl Microbiol 1975 29 807 813 16350018 10.1128/am.29.6.807-813.1975 PMC187084

[B45] Letort C Juillard V Development of a minimal chemically-defined medium for the exponential growth of *Streptococcus thermophilus* J Appl Microbiol 2001 91 1023 1029 11851809 10.1046/j.1365-2672.2001.01469.x

[B46] Green MR Sambrook J Molecular Cloning: A Laboratory Manual 2012 N. Y.: Cold Spring Harbor Laboratory Press

[B47] Untergasser A Nijveen H Rao X Bisseling T Geurts R Leunissen JAM Primer3Plus, an enhanced web interface to Primer3 Nucleic Acids Res 2007 35 W71 W74 17485472 10.1093/nar/gkm306 PMC1933133

[B48] Maguin E Prevost H Ehrlich SD Gruss A Efficient insertional mutagenesis in lactococci and other gram-positive bacteria J Bacteriol 1996 178 931 935 8550537 10.1128/jb.178.3.931-935.1996 PMC177749

[B49] Chopard MA Schmitt M Perrerad E Chamba JF Qualitative aspect of proteolytic activity of thermophilic lactobacilli using in Swiss cheeses Lait 2001 81 183 194

