# Supplementary material for: The naturally competent strain Streptococcus thermophilus LMD-9 as a new tool to anchor heterologous proteins on the cell surface
Source: Microb Cell Fact. 2014 Jun 5;13:82. doi: 10.1186/1475-2859-13-82 (PMC4076053; doi:10.1186/1475-2859-13-82)
Supplement: Additional file 1 — Proteins identified after cell surface shaving of S. thermophilus strains PrtS - , LMD-9, PrtH + and PrtH + WANS. [file 1475-2859-13-82-S1.pdf]

A. Proteins identified after cell surface shaving of *S. thermophilus* strains PrtS$^-$, LMD-9, PrtH$^+$ and PrtH$^+$WANS.

				PrtS$^-$		LMD-9		PrtH$^+$		PrtH$^+$WANS	
Description	Prot Id	MW (kDa)	Location	Nb id pept	emPAI	Nb id pept	emPAI	Nb id pept	emPAI	Nb id pept	emPAI
Subtilisin-like serine protease (PrtS)	Q03L35	172.9	CS			60	112.92				
PrtH$^+$		208.6	CS					40	6.86		
PrtH$^+$WANS		206.0	CS							82	161.66
Possible cell surface protein (STER_0576)	Q03LS6	108.3	CS	38	104.64	7	0.64	36	34.29	37	32.40
5'-nucleotidase/2'.3'-cyclic phosphodiesterase or related esterase (STER_0198)	Q03MQ2	91.1	CS	50	108 901.26	14	3.64	45	12914.50	47	39136.47
Surface antigen (STER_0478 = Cell separation envelope protein Cse)	Q03M12	50.4	CS	3	999.00	6	31621.78	5	31621.78	4	9999.00
Surface antigen (STER_0042)	Q03N07	46.4	CS	5	9.00	3	2.16	6	12.34	6	9.00
Aggregation promoting factor related surface protein (STER_1982)	Q03I85	19.8	CS	7	36.28	6	25.83			5	9.00
Enolase (eno)	*Q03LI0*	46.9	CS/Cyto			7	1.23	3	0.35		
ABC-type oligopeptide transport system (STER_1411)	Q03JP7	72.1	CS	25	42.94	29	33.33	24	23.71	47	9209.55
ABC-type oligopeptide transport system (STER_1409)	Q03JP9	71.7	CS	21	17.17	27	26.83	24	20.54	38	1290.55
Trypsin-like serine protease (STER_2002 = htrA)	Q03I68	42.7	CS					5	2.83	5	2.83
ABC-type Fe3+-hydroxamate transport system (STER_1025)	Q03KN8	37.9	CS	5	1.25					3	0.50
Uncharacterized ABC-type transport system (STER_0856)	Q03L27	37.5	CS			4	1.15			9	4.62
ABC-type amino acid transport/signal transduction system (STER_1539)	Q03JD1	31.2	CS					5	3.22		
ABC-type amino acid transport/signal transduction system (STER_1619)	Q03J56	30.6	CS	5	1.89						
ABC-type amino acid transport/signal transduction system (STER_1455)	Q03JK6	29.3	CS					3	0.70	5	1.42
ABC-type amino acid transport/signal transduction system (STER_1452)	Q03JK9	28.2	CS							4	1.03
Na+/xyloside symporter or related transporter (STER_1367)	Q03JT2	69.1	M			3	0.64				
High-affinity Fe2+/Pb2+ permease(STER_1022)	Q03KP1	61.0	M	6	1.96	9	6.63	8	2.38	10	7.73

Pyruvate-formate lyase(STER_1622)	Q03J53	86.9	Cyto			3	0.21				
ATP-binding subunit of Clp protease and DnaK/DnaJ chaperones (STER_1578)	Q03J94	77.0	Cyto	13	1.83	13	2.81	19	4.12	4	0.35
Elongation factor G (fusA)	*Q03IS1*	76.5	Cyto					8	0.95		
60 kDa chaperonin (groL)	*Q03MK3*	56.8	Cyto			6	0.74				
Transcriptional regulator (STER_1071)	Q03KK2	53.4	Cyto	6	2.16					3	0.67
SSU ribosomal protein S1P (STER_0639)	Q03LL8	43.8	Cyto					8	2.59		
Transcriptional regulator (STER_0378)	Q03M93	43.8	Cyto	5	4.01			4	1.51		
SSU ribosomal protein S1P (STER_0639)	Q03LL8	43.8	Cyto			4	0.90				
Elongation factor Tu (tuf)	*Q03LX0*	43.7	Cyto	11	5.95	13	17.33	12	9.00	11	3.83
Phosphoglycerate kinase(pgk)	*Q03IS8*	42.1	Cyto			3	0.41				
50S ribosomal protein L2 (rplB)	*Q03IF4*	29.9	Cyto	4	2.59			7	11.92		
30S ribosomal protein S3 (rpsC)	*Q03IF7*	24.0	Cyto					3	1.15		
Protein GrpE (grpE)	*Q03MR7*	23.3	Cyto					4	4.01		
30S ribosomal protein S4 (rpsD)	*Q03I94*	23.0	Cyto	3	0.64						
50S ribosomal protein L4 (rplD)	*Q03IF2*	22.1	Cyto	3	2.16			4	9.00		
50S ribosomal protein L5 (rplE)	*Q03IG3*	19.7	Cyto					5	2.51		
Heat shock protein Hsp20	Q03I62	16.3	Cyto					4	9.00		
50S ribosomal protein L17 (rplQ)	*Q03IH8*	14.4	Cyto					4	24.12		
50S ribosomal protein L7/L12 (rplL)	*Q03LT1*	12.3	Cyto	3	1.37	3	2.16	4	2.16		
Phosphotransferase system. HPr-related protein (STER_1243)	Q03K41	8.8	Cyto					3	99.00		
50S ribosomal protein L29 (rpmC)	*Q03IF9*	7.8	Cyto					3	30.62		
50S ribosomal protein L30 (rpmD)	*Q03IG9*	6.3	Cyto					3	315.23		
Uncharacterized conserved protein (STER_0283)	Q03MH9	66.0	-	3	0.78			3	0.33	4	0.62
Uncharacterized conserved protein (STER_0734)	Q03LD2	46.0	-	8	6.50			8	5.49	6	2.65
Uncharacterized protein (STER_0708)	Q03LF8	21.9	-	3	1.37						
Uncharacterized conserved phage related protein (STER_0813)	Q03L63	18.8	-	3	2.98					6	38.81

emPAI: exponentially modified Protein Abundance Index [31]

Nb id pept: number of identified peptides belonging to identified protein

Prot Id: Identification sequence from the following databases TRembl or Swiss prot (in italic)

Locations of proteins were predicted either by Uniprot data bank or by similarity to subcellular located proteins [35]. Three locations are proposed: cell surface (CS), membrane-located protein (M) and cytoplasm (Cyto).

B. Identified peptides of PrtS cell envelope proteinase (CEP) after shaving

Peptide		Sequence	Length	CEP Domains
Start	End			
158	176	LSVADETTAITNQEEAKPQ	19	Propeptide domain
158	188	LSVADETTAITNQEEAKPQNIDSNTIITVPK	31	Propeptide/Catalytic
177	188	NIDSNTIITVPK	12	Catalytic domain
196	224	GEGTVVAIIDSGLDVDHDVLHISDLSTAK	29	Catalytic domain
216	224	HISDLSTAK	9	Catalytic domain
236	249	EVAGISYGEWFNDK	14	Catalytic domain
253	264	GYNYVDVNTVLK	12	Catalytic domain
312	321	ATTGAALYVK	10	Catalytic domain
329	360	LGADSINLSLGGANGSVVNMNENVTAAIEAAR	32	Catalytic domain
350	360	ENVTAAIEAAR	11	Catalytic domain
362	400	AGVSVVIAAGNDGTFGSGHSNPSADYPDYGLVGAPSTAR	39	Catalytic domain
381	400	SNPSADYPDYGLVGAPSTAR	20	Catalytic domain
401	416	DAISVASYNNTTVGSK	16	Catalytic domain
417	433	VINIIGLENNADLNYGK	17	Catalytic domain
420	433	IIGLENNADLNYGK	14	Catalytic domain
421	433	IGLENNADLNYGK	13	Catalytic domain
424	433	ENNADLNYGK	10	Catalytic domain
451	473	EYEYVYAGIGQASDFDGLDLTGK	23	Catalytic domain
463	473	SDFDGLDLTGK	11	Catalytic domain
615	624	QYLQATYPTK	10	Catalytic domain
616	624	YLQATYPTK	9	Catalytic domain
624	634	KSPQEIEALVK	11	Catalytic domain
625	634	SPQEIEALVK	10	Catalytic domain
648	656	ETTAYTSPR	9	Catalytic domain
708	730	TLNYSTQLTTDTVQNGLITLAPR	23	A domain
711	730	YSTQLTTDTVQNGLITLAPR	20	A domain
712	730	STQLTTDTVQNGLITLAPR	19	A domain
716	730	TTDTVQNGLITLAPR	15	A domain
722	730	NGLITLAPR	9	A domain
769	778	NGYYLEGFVR	10	A domain
779	795	FTDVADGGDIVSIPYIG	17	A domain
779	797	FTDVADGGDIVSIPYIGFR	19	A domain
798	818	GEFQNLAVLEEPIYNLIADGK	21	A domain
807	818	EEPIYNLIADGK	12	A domain
819	856	GGFYFEPVTAQPDSVDISHHYTGLVTGSTELIYSTDKR	38	A domain
870	903	NEAGYFVLELDESGKPHLAISPNGDDNQDSLAFK	34	A domain
876	903	VLELDESGKPHLAISPNGDDNQDSLAFK	28	A domain
893	903	GDDNQDSLAFK	11	A domain
909	925	NYTDLVASVYAADDTER	17	A domain

926	939	TNPLWESQPQSGNK	14	A domain
951	973	SSIIYPTEWNGTDSEGNALADGK	23	A domain
953	973	IIYPTEWNGTDSEGNALADGK	21	A domain
958	973	EWNGTDSEGNALADGK	16	A domain
1001	1020	ESPVITTATYDETNFTFNPR	20	A domain
1033	1063	EQVFYLVADASGVTTIPSLLENGDVTVSDNK	31	A domain
1064	1085	VFVAQNDDGSFTLPLDLADISK	22	A domain
1086	1101	FYYTVEDYAGNISYEK	16	A domain
1087	1101	YYTVEDYAGNISYEK	15	A domain
1088	1101	YTVEDYAGNISYEK	14	A domain
1102	1112	VENLISIGNEK	11	A/H domains
1123	1142	DTNSPVPILFSYSVTDETGK	20	H domain
1132	1142	FSYSVTDETGK	11	H domain
1133	1142	SYSVTDETGK	10	H domain
1150	1158	YAGDTSVLK	9	H domain
1169	1183	FLYDTEWSSLAGETK	15	H domain
1170	1183	LYDTEWSSLAGETK	14	H domain
1228	1239	ADGQAIQLPNAK	12	H domain
1294	1307	ELIAELAGLEETAR	14	H domain
1322	1333	ALEDANAVYANK	12	H domain
1363	1386	LIAEVSNYTPTQANFIYYNAENTK	24	H domain

Cell surface proteins of *S. thermophilus* wild-type LMD-9 were shaved by trypsin.

Peptides corresponding to CEP of the wild-type strain LMD-9 are presented with their start and end on the protein including the signal sequence. Their sequence, length and the corresponding domain are indicated.

C. Identified peptides of PrtH+ cell envelope proteinase (CEP) after shaving

Peptide		Sequence	Length	CEP Domains
Start	End			
56	67	AAESTTANLTNK	12	Propeptide domain
205	224	YKGEGTVVSIIDTGIDPNHK	20	Catalytic domain
207	224	GEGTVVSIIDTGIDPNHK	18	Catalytic domain
339	367	LGADVLNMSLGSVSGEQTEDDPEVAAVER	29	Catalytic domain
371	394	KGTAAVISAGNSGTSNSEIEGVNK	24	Catalytic domain
395	412	AYYGNPDMETLGNPGTAR	18	Catalytic domain
397	412	YGNPDMETLGNPGTAR	16	Catalytic domain
413	424	SATTVASAENTK	12	Catalytic domain
425	438	ATTDGVTITSADGK	14	Catalytic domain
439	455	TTIAGPEATQLSEGTDR	17	Catalytic domain
505	520	YAQEAGAAGLIIVNNK	16	Catalytic domain
521	545	AGDITGMLLNAGFPTAGLSATSGEK	25	Catalytic domain
549	559	YVEAHPDEALK	11	Catalytic domain
560	572	VSIVVQALNNSAR	13	Catalytic domain
639	648	NGAFYATYQK	10	Catalytic domain
660	682	TLEMNTASIQPDISHDNVIVSPR	23	Catalytic domain
684	700	QGAGFINANATIQALAK	17	Catalytic/A domains
701	717	NPSTVVSSNGYPGVELK	17	A domain
748	765	NSDVYTSATDSSAVLYDK	18	A domain
748	766	NSDVYTSATDSSAVLYDKK	19	A domain
774	786	ASGDIVVPANSTK	13	A domain
787	799	ELTLTLTLPSDFK	13	A domain
800	819	ENQYVEGFLTFNSSDSSQLR	20	A domain
922	934	VINTLASLSNATK	13	A domain
935	964	TYYNSQAQSYTYFDDAPSWDGTYFDQQANK	30	A domain
965	976	TVNAPDGNYTYR	12	A domain
977	995	ISATIDGTNTEQHYDIPVK	19	A domain
1137	1149	NGTYVFSGTYPSK	13	B domain
1158	1172	DGQTHDLNVESDGNK	15	B domain
1187	1198	TTVTLYADSDHK	12	B domain
1203	1214	KQDITVSLVPAK	12	B domain
1231	1245	DSSAALAQTSENTVK	15	B domain
1271	1307	LNADHTFSTELPVSFGENDFTIVATDSNGNSSSVEQK	37	B domain
1316	1330	TTVSSSDVTFDNGIK	15	B domain
1380	1390	VVLNIGTHGAK	11	B domain
1391	1402	IFPALIGDSTVR	12	B domain

1433	1446	DKFTISGTISDDYK	14	B domain
1447	1469	FYDLSINGNDVETSWSAVDYHSK	23	B domain
1824	1833	STAQAYSSLK	10	W domain (S-Layer)
1876	1885	NSFVYQSNGK	10	W domain (S-Layer)

Cell surface proteins of *S. thermophilus* LMD-9 PrtH$^+$ mutant were shaved by trypsin.

Peptides corresponding to CEP of PrtH$^+$ mutant are presented with their start and end on the protein including the signal sequence. Their sequence, length and the corresponding domain are indicated.

D. Identified peptides of PrtH^{+}WANS cell envelope proteinase (CEP) after shaving

Peptide		Sequence	Length	CEP Domains
Start	End			
56	67	AAESTTANLTNK	12	Propeptide domain
100	124	SATPAATNGSVSANSSSAEIEQASK	25	Propeptide domain
148	159	SYGYVVNGFATK	12	Propeptide domain
182	204	VYYANDSSADNMANVSTVWNNYK	23	Propeptide/Catalytic
205	224	YKGEGTVVSIIDTGIDPNHK	20	Catalytic domain
314	338	AFSNSDSSASTDSTSIIGAIDDSAK	25	Catalytic domain
339	367	LGADVLNMSLGSVSGEQTEDDPEVAAVER	29	Catalytic domain
347	367	SLGSVSGEQTEDDPEVAAVER	21	Catalytic domain
371	394	KGTAAVISAGNSGTSNSEIEGVNK	24	Catalytic domain
372	394	GTAAVISAGNSGTSNSEIEGVNK	23	Catalytic domain
395	412	AYYGNPDMETLGNPGTAR	18	Catalytic domain
397	412	YGNPDMETLGNPGTAR	16	Catalytic domain
413	424	SATTVASAENTK	12	Catalytic domain
425	438	ATTDGVTITSADGK	14	Catalytic domain
439	455	TTIAGPEATQLSEGTDR	17	Catalytic domain
521	545	AGDITGMLLNAGFPTAGLSATSGEK	25	Catalytic domain
549	559	YVEAHPDEALK	11	Catalytic domain
560	572	VSIVVQALNNSAR	13	Catalytic domain
626	638	GTQALVSQTMNDK	13	Catalytic domain
639	648	NGAFYATYQK	10	Catalytic domain
660	675	TLEMNTASIQPDISHD	16	Catalytic domain
660	682	TLEMNTASIQPDISHDNVIVSPR	23	Catalytic domain
667	682	SIQPDISHDNVIVSPR	16	Catalytic domain
684	700	QGAGFINANATIQALAK	17	Catalytic/A domains
699	717	AKNPSTVVSSNGYPGVELK	19	A domain
701	717	NPSTVVSSNGYPGVELK	17	A domain
703	717	STVVSSNGYPGVELK	15	A domain
748	765	NSDVYTSATDSSAVLYDK	18	A domain
748	766	NSDVYTSATDSSAVLYDKK	19	A domain
754	765	SATDSSAVLYDK	12	A domain
754	766	SATDSSAVLYDKK	13	A domain
774	786	ASGDIVVPANSTK	13	A domain
787	799	ELTLTLTLPSDFK	13	A domain
800	810	ENQYVEGFLTF	11	A domain
800	819	ENQYVEGFLTFNSSDSSQLR	20	A domain
805	819	EGFLTFNSSDSSQLR	15	A domain
809	819	TFNSSDSSQLR	11	A domain
810	819	FNSSDSSQLR	10	A domain
922	934	VINTLASLSNATK	13	A domain

935	964	TYYNSQAQSYTYFDDAPSWDGTYFDQQANK	30	A domain
940	964	QAQSYTYFDDAPSWDGTYFDQQANK	25	A domain
943	964	SYTYFDDAPSWDGTYFDQQANK	22	A domain
965	976	TVNAPDGNYTYR	12	A domain
966	976	VNAPDGNYTYR	11	A domain
967	976	NAPDGNYTYR	10	A domain
977	995	ISATIDGTNTEQHYDIPVK	19	A domain
977	1004	ISATIDGTNTEQHYDIPVKVDSVAPVVK	28	A domain
981	995	IDGTNTEQHYDIPVK	15	A domain
985	995	NTEQHYDIPVK	11	A domain
1032	1059	DELSGLSGDANVSVNGVSAQLEYDPTAK	28	A domain
1068	1078	VEIDLSPAQAK	11	A domain
1079	1118	ALQAGTNTFSVALFDNAANAGTASGEGNKPGETNFGLVLR	40	A/B domains
1088	1118	SVALFDNAANAGTASGEGNKPGETNFGLVLR	31	A/B domains
1137	1149	NGTYVFSGTYPSK	13	B domain
1150	1172	LYGTYTDKDGQTHDLNVESDGNK	23	B domain
1158	1172	DGQTHDLNVESDGNK	15	B domain
1187	1198	TTVTLYADSDHK	12	B domain
1203	1214	KQDITVSLVPAK	12	B domain
1204	1214	QDITVSLVPAK	11	B domain
1215	1230	VESLSVDKNDTYDETK	16	B domain
1215	1245	VESLSVDKNDTYDETKDSSAALAQTSENTVK	31	B domain
1231	1245	DSSAALAQTSENTVK	15	B domain
1271	1307	LNADHTFSTELPVSFGENDFTIVATDSNGNSSSVEQK	37	B domain
1314	1330	GKTTVSSSDVTFDNGIK	17	B domain
1316	1330	TTVSSSDVTFDNGIK	15	B domain
1356	1368	VKRPTTTLQIGGK	13	B domain
1380	1390	VVLNIGTHGAK	11	B domain
1391	1402	IFPALIGDSTVR	12	B domain
1409	1432	LSFYVDAEAPTLNLDSENTVYTNK	24	B domain
1433	1446	DKFTISGTISDDYK	14	B domain
1435	1446	FTISGTISDDYK	12	B domain
1447	1467	FYDLSINGNDVETSWSAVDYH	21	B domain
1447	1469	FYDLSINGNDVETSWSAVDYHSK	23	B domain
1475	1483	NFKHEVDLK	9	B domain
1493	1513	VTDIQGNSSSQALVVYYEPAK	21	B domain
1514	1526	TLAEPSVEQVVNK	13	B domain
1564	1573	VTENGTVQFK	10	B domain
1828	1851	KDEGTVTPPPIDSEIVDVQAPPVK	24	W domain (PrtS)
1829	1851	DEGTVTPPPIDSEIVDVQAPPVK	23	W domain (PrtS)
1852	1873	DTGNSEHVPIGQKPNPQPTLPR	22	W domain (PrtS)
1852	1879	DTGNSEHVPIGQKPNPQPTLPRPVTLQA	28	W domain (PrtS)
1874	1888	PVTLQASLSSPNQEK	15	W domain (PrtS)

Cell surface proteins of *S. thermophilus* LMD-9 PrtH$^+$WANS mutant were shaved by trypsin.
Peptides corresponding to CEP of the PrtH$^+$WANS mutant are presented with their start and end on the protein including the signal sequence. Their sequence, length and the corresponding domain are indicated.
