# Supplementary material for: The naturally competent strain Streptococcus thermophilus LMD-9 as a new tool to anchor heterologous proteins on the cell surface
Source: Microb Cell Fact. 2014 Jun 5;13:82. doi: 10.1186/1475-2859-13-82 (PMC4076053; doi:10.1186/1475-2859-13-82)
Supplement: Additional file 3 — Primers used for qPCR experiments. [file 1475-2859-13-82-S3.pdf]

Primers used for qPCR experiments

qPCR Primer	Sequence	Size (bp)	Hyb Temp (°C)	DNA target	Product Size (bp)
Sigmaq_F	GACCCAACACCTGAGCAAAT	20	58	*sigma70/32*	130
Sigmaq_R	CCAAATGGCTGTCATCCTCT	20			
PrtSq_F	AATGCTTTAGCAGATGGTAAGT	22	58	*prtS*	126
PrtSq_R	CATAGGTAGCTGTGGTGATAA	21			
PrtHq_F	TGTAGCAACCGACTCTAATGG	21	58	*prtH*	133
PrtHq_R	CGAGTACCCCACTTGATACC	20			

The name, sequence, size, hybridization temperature (Hyb Temp), DNA target and expected product size are indicated.
bp (base pairs)
